# PD214 Appraisal Of Reimbursement Thresholds For Medicines In Brazil’s Private Health System

**DOI:** 10.1017/S0266462324004355

**Published:** 2025-01-07

**Authors:** Marcus Carvalho Borin, Carina Rejane Martins, Daniel Pitchon dos Reis, Geraldo Jose Coelho Ribeiro, Julia Teixeira Tupinambas, Karina de Castro Zocrato, Lelia Maria de Almeida Carvalho, Marcela Pinto de Freitas, Maria da Gloria Cruvinel Horta, Mariana Michel Barbosa, Mariza Cristina Torres Talim, Sergio Adriano Loureiro Bersan, Silvana Marcia Bruschi Kelles

## Abstract

**Introduction:**

Brazil’s public health system serves most of the population, but 25 percent of citizens rely on private health insurance. The National Regulatory Agency for Private Health Insurance and Plans (ANS) regulates private medicine reimbursements, which diverge from the public sector threshold. In 2022, the National Committee for Health Technology Incorporation (CONITEC) set a willingness-to-pay benchmark of BRL40,000 (USD8,215) per quality-adjusted life-year. The ANS has no such benchmark, highlighting a pivotal gap in economic evaluations for private health care.

**Methods:**

This quantitative study investigated the Incremental cost-effectiveness ratios (ICER) for reimbursed medicines in Brazil’s private health sector, comparing them with CONITEC’s benchmarks and international thresholds. Data were extracted from industry reimbursement submissions to the ANS and analyzed for statistical disparity and policy implications.

**Results:**

Preliminary findings found an ICER peak of BRL619,900 (USD127,220) per quality-adjusted life-year for talazoparib, which is used to treat certain advanced breast cancers. This contrasted sharply with CONITEC’s established threshold, indicating a critical need to evaluate ANS policies.

**Conclusions:**

Early results indicate that the ICERs for some medicines surpass CONITEC’s willingness-to-pay limit, suggesting that the ANS should consider establishing a defined cost-effectiveness threshold. This is imperative to harmonize with global standards and maintain sustainable health financing.

